# A Novel G-Protein-Coupled Receptors Gene from Upland Cotton Enhances Salt Stress Tolerance in Transgenic *Arabidopsis*

**DOI:** 10.3390/genes9040209

**Published:** 2018-04-12

**Authors:** Pu Lu, Richard Odongo Magwanga, Hejun Lu, Joy Nyangasi Kirungu, Yangyang Wei, Qi Dong, Xingxing Wang, Xiaoyan Cai, Zhongli Zhou, Kunbo Wang, Fang Liu

**Affiliations:** 1 Research Base in Anyang Institute of Technology, State Key Laboratory of Cotton Biology, Institute of Cotton Research, Chinese Academy of Agricultural Science (ICR, CAAS), Anyang 455000, Henan, China; lupu1992@cricaas.com.cn (P.L.); magwangarichard@yahoo.com (R.O.M.); joynk@cricaas.com.cn (J.N.K.); dongqi@cricaas.com.cn (Q.D.); wangxx@cricaas.com.cn (X.W.); caixy@cricaas.com.cn (X.C.); zhouzl@cricaas.com.cn (Z.Z.); 2 Jaramogi Oginga Odinga University of Science and Technology, P.O. Box 210-40601, 210 Bondo, Kenya; 3 Gembloux Agro-Bio Tech, University of Liège, 5030 Gembloux, Belgium; hejunlu@cricaas.com.cn; 4 Anyang Institute of Technology, Anyang 455000, Henan, China; weiyangyang@cricaas.com.cn

**Keywords:** G-protein-coupled receptors, salt tolerance, antioxidant, gene, wild type, transgenic

## Abstract

Plants have developed a number of survival strategies which are significant for enhancing their adaptation to various biotic and abiotic stress factors. At the transcriptome level, G-protein-coupled receptors (GPCRs) are of great significance, enabling the plants to detect a wide range of endogenous and exogenous signals which are employed by the plants in regulating various responses in development and adaptation. In this research work, we carried out genome-wide analysis of target of Myb1 (*TOM1*), a member of the GPCR gene family. The functional role of TOM1 in salt stress tolerance was studied using a transgenic *Arabidopsis* plants over-expressing the gene. By the use of the functional domain PF06454, we obtained 16 *TOM* genes members in *Gossypium hirsutum*, 9 in *Gossypium arboreum*, and 11 in *Gossypium raimondii*. The genes had varying physiochemical properties, and it is significant to note that all the grand average of hydropathy (GRAVY) values were less than one, indicating that all are hydrophobic in nature. In all the genes analysed here, both the exonic and intronic regions were found. The expression level of *Gh_A07G0747 (GhTOM)* was significantly high in the transgenic lines as compared to the wild type; a similar trend in expression was observed in all the salt-related genes tested in this study. The study in epidermal cells confirmed the localization of the protein coded by the gene *TOM1* in the plasma membrane. Analysis of anti-oxidant enzymes showed higher concentrations of antioxidants in transgenic lines and relatively lower levels of oxidant substances such as H_2_O_2_. The low malondialdehyde (MDA) level in transgenic lines indicated that the transgenic lines had relatively low level of oxidative damage compared to the wild types. The results obtained indicate that *Gh_A07G0747 (GhTOM)* can be a putative target gene for enhancing salt stress tolerance in plants and could be exploited in the future for the development of salt stress-tolerant cotton cultivars.

## 1. Introduction

Because plants are sessile, they are strongly affected by the ever-deteriorating environmental conditions [1]. Plants have developed a number of survival strategies which are significant for enhancing their adaptation to various biotic and abiotic stress factors [2]. Being that abiotic stresses are dynamic and complex in nature, plants have developed complex mechanisms which encompass changes at the transcriptome, cellular, and physiological levels [3]. At the transcriptome level, G-protein-coupled receptors (GPCRs) are of great significance, enabling the plants to detect a wide range of endogenous and exogenous signals employed by the plants in the regulation of various responses in development and adaptation [4]. The external signals include all the abiotic and biotic factors which have direct effects on the plants’ growth and development, such as light, temperature, mechanical perturbations (e.g., wind), humidity, CO_2_, edaphic factors (e.g., soil water and nutrients), and gravity. The internal signals mainly include growth regulators, developmental regulators, and metabolites [5]. When plants are exposed to primary stimuli such as water deficit, salt stress, light, hormones, and pathogen-derived elicitors, the primary stimuli can cause membrane depolarization of the plant cells within a few seconds, as a result of early Ca^2+^ influx and anion efflux through ion channels [6].

G-protein-coupled receptors are protein domains with seven putative transmembrane segments (7TM), and are mainly responsible for transmitting extracellular signals to the intracellular region as well as triggering several signalling cascades in response to stimuli as diverse as light, protons, Ca^2+^, odorants, amino acids, nucleotides, proteins, peptides, steroids, and fatty acids [7]. The GPCRs play key roles in cellular transduction, and hence are considered as critical regulators in cellular processes. Moreover, the fact that their dysfunction can result in several diseases has made GPCRs the targets of the majority of the currently-prescribed drugs [8]. It seems that the signals are mostly perceived at the level of membrane, and therefore transmembrane events are the likely routes for signal generation and transduction. In plants, the best-characterized plasma membrane-based receptors are of two kinds: (i) transmembrane receptor enzymes (usually kinase); and (ii) G-protein-coupled receptors. Over the last few years, a number of receptors have been identified in plants, which has helped in understanding the plant signal transduction network. Presently, in plants, the G-proteins are reported to be involved in processes such as ion channel and abscisic acid signalling [9] and modulation of cell proliferation in *Arabidopsis*. However, a wide range of processes—including seed germination, shoot and root growth, and stomatal regulation—are altered in *Arabidopsis* and rice plants with mutations in G-protein components. Furthermore the GPCR proteins have been found to function in gibberellic acid (GA) pathways and in some developmental responses [10]. Recently, the role of heterotrimeric G proteins G*α* in pathogenicity [11] and biotic stresses [12] has also been reported.

The GPCRs are significantly important to drug industry application in humans and other animals, while little is known about plant GPCRs. Usually, the identification of new GPCRs from genome-wide analysis enables the prediction of novel important receptors in a number of species, while the identification of GPCRs in plants are still debatable compared to animal GPCRs [13]. The main reason for the doubt of the existence of GPCRs in plants is because of the functional paradigm of the animal kingdom, where the activity of GPCRs enables the development of complex signalling and sensing mechanisms, yet this is different in plants [14,15]. Compared to the animal kingdom where thousands of GPCRs have been identified, only a single GPCR has been clearly identified in plant system so far. In *Arabidopsis*, the gene corresponding to the canonical GPCR candidate was isolated by Josefsson and Rask [16] and Plakidou-Dymock et al. [17]. In their studies, they concluded that glycolysis regulation (*GCR)*1 encodes the first 7TM receptor homologue identified in higher plants, and is involved in cytokinin signal transduction. Recently, unique GPCR types have been identified in *Arabidopsis*, such as target of Myb1 (*TOM*)1 (*At4g21790.1*) and *TOM3* (*At2g02180.1*), all belonging to the family class A, sub-family Olfactory, but falling in different sub-family types Olfactory II family 10 and Olfactory II family 5, respectively [18]. The two GPCR candidate genes, *TOM1* and *TOM3,* have been found not to interact with guanine nucleotide-binding protein α-1 subunit (GPA1) compared to other functional forms of GPCR such as cullin associated and neddylation dissociated (CAND)1, 2, 3, 5, 7, 8 and heptahelical transmembrane protein (HHP)2, and therefore it has been proved that the inability of a particular gene to bind with Gα does not mean they are not members of GPCR so long as they are able to stimulate the exchange of guanosine diphosphate for guanosine triphosphate [18]. In addition, sequence comparison between the different GPCRs both in animals and plants have revealed the existence of different receptor families sharing no sequence similarity. However, all of these receptors have in common a central core domain constituted of seven transmembrane helices connected by three intracellular and three extracellular loops [19]. Two cysteine residues, which are conserved in most of the GPCRs, form a disulfide link which is probably important for the packing and for the stabilization of a restricted number of conformations of these seven transmembranes (TMs). Aside from sequence variations, GPCRs differ in the length and function of their N-terminal extracellular domain, their C-terminal intracellular domain, and their intracellular loops [20,21].

In the last decade, the physiological effect of GCR1 has been investigated in relation to seed germination, cell signalling mediators, response to both gibberellins and brassinosteroid, and even their possible role in enhancing drought stress tolerance [22,23]. Plants known to possess some levels of salt tolerance mainly depend on the cell membrane stability in order to sustain the selective uptake of ions and the absorption of water through osmosis [24]. Salt stress results in a range of responses in plants, while extreme salinity may result in oxidative damage, ion toxicity, nutrition imbalance, and eventually plant death [25]. Plants do produce reactive oxygen species (ROS) during metabolic activities in the form of singlet oxygen (O^2^_1_), hydrogen peroxide (H_2_O_2_), hydroxyl radical (HO^−^), and superoxide radical (O_2_^−^) [26]. The ROS are generated and eliminated by the plants in a synchronized mechanism, but when plants are exposed to a stress factor, the system shuts down, resulting in imbalance. Thus, the excessive production of ROS by the plants results in the synthesis of some vital antioxidants, such as superoxide dismutase (SOD), peroxidase (POD), polyphenol oxidase (PPO), chloramphenicol acetyltransferase (CAT), and malondialdehyde (MDA), which are vital for DNA repair to maintain normal growth during stress conditions [27]. The injuries caused by an increased threshold of ROS is known as oxidative stress. ROS can cause oxidative damage to various cellular components, such as membrane lipids, proteins, nucleic acids, and chlorophyll [28]. The effect of oxidative stress has been reported on cotton [29], rice [30], wheat, and beans [31]. ROS cause chlorophyll degradation and membrane lipid peroxidation, reducing membrane fluidity and selectivity [32]. The chlorophyll loss and lipid peroxidation are quantified by the MDA concentration within the cell, as MDA is the product of lipid peroxidation [33].

In this research work, we transformed a novel *Gossypium hirsutum* GPCR candidate gene, *Gh_A07G0747* (*GhTOM*), into the model plant *Arabidopsis thaliana*, and carried out the functional and expression analysis under salt stress condition. The findings provide significant information on the potential role of the novel gene designated as *Gh_A07G0747* (*GhTOM*) from *G. hirsutum* in enhancing salt stress tolerance in transgenic *Arabidopsis,* and therefore suggest its suitability for future molecular functional characterization and application in the development of cotton genotypes with enhanced salt-stress-tolerance.

## 2. Materials and Methods

### 2.1. Identification and Sequence Analysis of TOM Proteins in Cotton

The three cotton genome sequences were downloaded from three different cotton genome websites: *Gossypium hirsutum* TOM protein sequences were downloaded from the Cotton Research Institute (http://mascotton.njau.edu.cn, Nanjing, China), *Gossypium arboreum* were obtained from the Beijing Genome Institute (https://www.bgi.com/, Beijing, China), and *Gossypium raimondii* genome from Phytozome 12 (http://www.phytozome.net/, Joint Genome Institute, Department of Energy), with E-value < 0.01. The conserved domain of TOM protein (PF06454) was downloaded from Pfam protein families (http://pfam.xfam.org/, European Molecular Biology Laboratory). For the identification of TOM proteins in cotton, the hidden Markov model analysis (HMM) profile of TOM protein was used as a query to carry out the HMMER search (http://hmmer.janelia.org/) [34] against *Gossypium hirsutum*, *Gossypium raimondii*, and *Gossypium arboreum*. In order to confirm the presence of the TOM domain, two online tools were later used for further analysis: the ScanProsite tool (http://prosite.expasy.org/scanprosite/, Swiss Institute of Bioinformatics) and the Simple Modular Architecture Research Tool (SMART) program (http://smart.embl-heidelberg.de/). SMART and Pfam databases were used to verify the presence of the *TOM* gene domains. The isoelectric points (PIs) and molecular weights of TOM proteins were estimated by an online ExPASy Server tool (http://www.web.xpasy.org/compute_pi/, Swiss Institute of Bioinformatics). In addition, Wolfpsort (https://www.wolfpsort.hgc.jp/) [35] was used to determine the subcellular location of all the TOM proteins within the cell. The results obtained for the subcellular location for the all the TOM proteins were further validated by using two other online tools: TargetP1.1 server [36] and Protein Prowler Subcellular Localisation Predictor version 1.2 (http://www.bioinf.scmb.uq.edu.au/pprowler_webapp_1-2/) [37]. In addition, *Arabidopsis thaliana* TOM protein sequences were downloaded from the *Arabidopsis* genome sequencing resource, the *Arabidopsis* information resource (TAIR) (http://www.arabidopsis.org/).

### 2.2. Chromosomal and Subcellular Localization of the TOM Protein in Cotton

The chromosome locations of all the *TOM* genes as obtained for all the three cotton genomes were determined through a blast search in Cotton Functional Genome Database [38], using their respective gene identities. In addition, we determined the subcellular localization of the *TOM* genes in cotton through the online software Wolfpsort (https://wolfpsort.hgc.jp/) and validated the results through TargetP1.1 [36] and Protein Prowler Subcellular Localisation Predictor version 1.2, as used in the analysis of other cotton functional genes [39]. 

### 2.3. Phylogenetic Analysis and Functional Classification of TOM Proteins in Cotton 

To characterize the evolutionary features of TOM proteins, we used the gene identities of all the *TOM* genes, which we employed in downloading their respective protein sequences from the Cotton Functional Genome Database. The cotton *TOM* genes were analysed together with the sequences obtained from Phytozome for *Oryza sativa*, *Arabidopsis thaliana*, *Populus trichocarpa*, and *Theobroma cacao*. The multiple alignments of the TOM conserved domains were conducted using ClustalW program, a functional tool in MEGA 7.0 software [40], with a gap open penalty of 10 and gap extension penalty of 0.2. Thereafter, a neighbour joining (NJ) tree was performed with support for each node, tested with 1000 bootstrap replicates. The gene structures were obtained by comparing the genomic sequences and their predicted coding sequences from the cotton genome project and the structure displayed by the use of gene structure displayer server [41]. The protein sequences were analysed for motif identification by the Multiple EM for Motif Elicitation (MEME) program [42], with the default parameters adjusted to maximum number of motifs 20 and optimum motif width range set to >6 and <50.

### 2.4. Analysis of the Expression Patterns of Upland Cotton TOM Genes in Different Tissues, under Salt Stress through RNA Sequencing Data

This was performed in order to get the best sets of *TOM* genes for cloning. We obtained the RNA sequences data from the Cotton Functional Genome Database. The RNA-sequence data analysed were from torus, stamen, root, leaf, calycle, petal, pistil, and stem. Moreover, we investigated the expression levels of upland cotton *TOM* genes in response to the salt stress. Salt stress is among the major abiotic stress factors which has a profound effect on cotton production. The *Gh_A07G0747* gene (*TOM1*) was found to be the most highly upregulated gene across various tissues studied in this work (Supplementary Figure S1), and hence the gene was selected for a detailed functional characterization.

### 2.5. Plant Transformation and Screening of Gh_A07G0747 (GhTOM) Gene Arabidopsis Lines

In this research work, we used two main plants—namely the model plant *Arabidopsis thaliana*, ecotype Colombia-0 (Col-0). Because the cloned gene is from *Gossypium hirsutum*, we used *Gossypium hirsutum*, accession number *G. hirsutum*-CRI-12 (G09091801–2), in confirming the presence of the genes in various tissues. CRI-12 was developed by our research institute, Cotton Research Institute, an Institute of the Chinese Academy of Agricultural Science (CAAS). The *pWM101-35S:Gh_A07G0747* (*GhTOM*) construct in *Agrobacterium tumefaciens* GV3101 was confirmed by specific primers: the forward primer pair of *Gh_A07G0747* (*GhTOM*) (3′-CGGGATCCATGATTAAGTGTTACCCTTTC-5′) and reverse primer pair of *Gh_A07G0747* (*GhTOM*) (3′-GCTCTAGATTAATTTGGACTTGTTCTTG-5′) (Invitrogen, Beijing, China). Wild-type (WT) plants were transformed by use of floral dip method though with little modification [43]. Infiltration media used contained Murashige and Skoog (MS) medium 4.3 g/L, sucrose 50 g/L (5%), 2-(4-morpholino) ethane sulfonic acid (MES) 0.5 g/L, Silwet-77 200 µL/L (0.02%), 6-benzylaminopurine (6-BA) 0.01 mg/L with pH of 5.7. Transgenic lines were selected by germinating seeds on 0.5 Murashige and Skoog (MS) medium (PhytoTechnology Laboratories, Lenexa, KS, USA), containing 50 mg/L hygromycin B (Roche Diagnostics GmbH, Mannheim, Germany) for 3 days at 4 °C. This was to maximize germination by breaking seed dormancy, upon which the seedlings were transferred to an *Arabidopsis* growth chamber in a conditioned controlled room, in which the conditions were set at 16 h light/8 h dark. After one week in selection medium, at 3–4 true leaves emergence stage, the seedlings were transplanted into small pots filled with vermiculite and humus in the ratio of 1:1. The seedlings (T0) were grown to maturity to set seeds for second-generation (T1 seeds) in a well-conditioned *Arabidopsis* growth chamber. T1 seeds were germinated on antibiotics-selective medium, and the one-copy lines were singled out by examining the segregation ratio (3:1) of the antibiotics-selectable marker. T2 seeds were then germinated on antibiotics-selective medium again, and the one-copy lines were identified based on the seedling survival of the antibiotics-selectable marker. The T3 homozygous progeny was bred from a T2 population after real-time quantitative reverse transcription polymerase chain reaction (qRT-PCR) and the selection of three higher overexpression lines (OE-3, OE-7, and OE-9) using *Gh_A07G0747 (GhTOM)* forward primer (3′-TGCGAAAGCTTTTTCATCATTGG-5′) and *Gh_A07G0747 (GhTOM)* reverse primer (3′-ACTTGTAGACGGGGCTGGTA-5′) (Invitrogen, Beijing, China), with total complementary DNA (cDNA) as template. The phenotypic investigations were carried out on T3 homozygous lines. 

### 2.6. Subcellular Localization of Gh_A07G0747 (GhTOM) Protein

The open reading frame (ORF) of *Gh_A07G0747 (GhTOM)* without the stop codon was amplified by polymerase chain reaction (PCR) by the transformed gene specific primer. The forward primer sequence (*Gh_A07G0747 (GhTOM*) 3′-ACACGGGGGACTCTAGA**GGATCC**ATGATTAAGT GTTACCCTTT-5′ and reverse primer sequence *Gh_A07G0747 (GhTOM)* 3′-ACTCATACTAGTC CCGG**GGATCC**ATTTGGACTTGTTCTTGACAC-5′) (Invitrogen, Beijing, China) were obtained using proofreading *Pfu* DNA polymerase (TransGen Biotech, Beijing, China). The PCR products were then cloned into pBI121-GFP vector upstream of the green fluorescent protein (GFP) sequences producing the pBI121-Gh_A07G0747 (GhTOM)–GFP construct with *GhTOM–GFP* fusion gene under the regulation of CaMV 35S promoter. The cloning procedure was done as outlined in the kit manual, pEASY-Uni Seamless Cloning and Assembly Kit (TransGen Biotech, Beijing, China) [44,45]. The construct was then transferred into *Agrobacterium tumefaciens* strain LBA4404 (Shanghai Weidi Biotechnology Co., Shangai, China) and subsequently transformed into onion epidermal cells by adopting the method as outlined by Sun et al. [46]. The transformed onion epidermal cells were cultured on 0.5 MS media in a dark growth chamber for 20 h at a temperature of 25 °C. The expression of the genes transformed into the onion epidermal cells was observed using a Zeiss Model Axio Imager M1 Upright Fluorescent Microscope (430004-9901-Axio Imager.M1, Gottingen, Germany). 

### 2.7. Response of Overexpressed Gh_A07G0747 (GhTOM) Transgenic Lines to Salt Stress Tolerance

The T3 homozygous transgenic lines were sterilized by 10% bleach solution *(v*/*v)* for 10 min and washed three times with deionised and sterilized water. The sterilized seeds were then plated on 0.5 Murashige and Skoog medium. Plants were stratified at 4 °C in a dark chamber for a period of 3 days and then moved to a growth room with room temperature set at 22 °C with a 16 h light/8 h dark photoperiod. After one week, seedlings were transferred to small planting conical pots (bottom dimension 5 cm, top dimension 7 cm, depth 8 cm) containing well-watered mixtures of vermiculite and humus mixed in the ratio of 1:1, respectively. After 3 weeks of growth, the pots were subsequently watered every four days with water containing 0 mM NaCl, 150 mM NaCl, and 250 mM NaCl. The chlorophyll content determination was carried out after 8 days, while observation of the phenotypic traits was done at 16 days.

### 2.8. Root Elongation and Survival Assays

The three transgenic lines—overexpressed lines 3, 7, and 9, designated as OE-3, OE-7, and OE-9—were monitored for NaCl stress tolerance by first growing seedlings in vertical position in 0.5 MS for 5 days and then transferring to the new plates with 0.5 MS containing 0 mM NaCl, 150 mM, NaCl, and 200 mM NaCl concentrations; they were grown for 6 days. The root length was recorded on the seventh day post-transfer. Survival assay was carried out by using seeds from WT and *Gh_A07G0747 (GhTOM)* transgenic lines, sown in 0.5 MS for 5 days and then transferred to the new plates containing 0 mM and 250 mM NaCl concentrations. The plants’ survival rates were examined at the fourth day of germination.

### 2.9. Catalase Enzymes Extraction and Assay

Fresh leaf samples were frozen in liquid nitrogen upon harvesting from both treated and control plant samples. The frozen leaves were then kept at low temperature (−80 °C) for further analyses. For each sample, 0.5 g of leaf samples was vortexed with 5 mL of extraction buffer with 50 mM K-phosphate buffer with pH of 7.6 and 0.1 mM Na_2_EDTA (Amresco, Dallas, TX, USA). The buffer mixture was centrifuged at 15,000 rpm for 15 min, and the supernatant fraction was used to assay various enzymes. All steps in the preparation of enzyme extracts were performed at 4 °C. Catalase (CAT) activity was determined by monitoring the declining level of H_2_O_2_, as described by Cakmak and Marschner [47].

### 2.10. Superoxide Dismutase Enzymes Extraction and Assay

Three sets of leaf samples weighing 10 g each were obtained from both salt and control plants, and were frozen in liquid nitrogen upon collection. The leaf samples were crushed into fine powder on ice by the use of a mortar and pestle for a period of 2 min in 10 mL of homogenizing solution containing 50 mmol/L HEPES buffer and 0.1 mmol/L Na_2_EDTA (pH 7.6). The homogenate was centrifuged at 12,000 rpm for 20 min at a temperature of 4 °C, and the supernatant was used for superoxide dismutase (SOD) assays [48]. Superoxide dismutase activity was determined by monitoring the inhibition of the photochemical reduction of nitro blue tetrazolium (NBT), as described by Giannopolitis and Ries [49] with slight modifications. For the estimation of total SOD, a 5-mL reaction mixture was prepared, containing 50 mmol/L Na_2_CO_3_ (pH 10.4), 50 mM of HEPES (pH 7.6), 0.025% (*w*/*v*) Triton X-100, 75 μmol/L NBT, 0.1 mmol/L EDTA, 13 mmol/L methionine, 2 μmol/L riboflavin, and an aliquot of enzyme extract, and was illuminated for 10 min at a light intensity of 350 μmol/m^2^/s. A control reaction was maintained throughout, wherein all the steps and components were exactly the same as described above, except that crude enzyme was replaced with an equal volume of phosphate buffer (pH 7.8) [50]. A unit of SOD activity was evaluated as the amount of enzyme required to cause 50% inhibition of the reduction of NBT as monitored at 560 nm [51].

### 2.11. Chlorophyll Determination

Leaf sections weighing 200 mg were obtained from both treated and control samples, frozen in liquid nitrogen, then placed in 5 mL of absolute ethanol (99.9%) and heated in a water bath at 80 °C for 20 min. Total chlorophyll was evaluated in the alcohol extracts from absorbance readings, using the appropriate extinction coefficient. Chlorophyll content (mg/g fresh weight) was calculated as 100 × A_654_/(39.8 × sample fresh weight) as described by Tetley and Thimann [52].

### 2.12. Malondialdehyde Content

Lipid peroxidation was quantified as the amount of malondialdehyde (MDA) measured by the thiobarbituric acid (TBA) reaction [53]. Frozen samples were ground in a mortar and pestle with two volumes of ice-cold 0.1% (*w*/*v*) trichloroacetic acid (TCA) (Agrochemicals, Chennai, India) and centrifuged for 15 min at 15,000 rpm. The mixture containing 1 mL of the supernatant and 2 mL of 0.5% (*w*/*v*) TBA in 20% (*w*/*v*) TCA was heated at 95 °C for a half an hour and then rapidly cooled in an ice bath. After centrifugation at 12,000 rpm for 10 min at a temperature of 4 °C, the supernatant absorbance was read at 532 nm and the values corresponding to nonspecific absorption done at 600 nm.

### 2.13. qRT-PCR Analysis of the Expression of Salt-Responsive Genes in Transgenic Arabidopsis

To analyse the expression of *Gh_A07G0747 (GhTOM)* and the NaCl-responsive genes in the transgenic *Arabidopsis* plants, total RNA was isolated from four-week-old transgenic *Arabidopsis* seedlings and wild type (Columbia ecotype) grown under normal conditions (CK) and under 250 mM NaCl treatments for 4 days. The EASYspin Plus plant RNA extraction kit, obtained from Aid Lab (Beijing, China), was used to extract total RNA from the leaf tissues. The quality and concentration of each RNA sample was determined using gel electrophoresis and a NanoDrop 2000 spectrophotometer (Thermo Fisher, Waltham, MA, USA). Only RNAs which met the criterion 260/280 ratio of 1.8–2.1, 260/230 ratio ≥ 2.0 were used for further analyses, and were stored at −80 °C. The extracted RNAs were reverse-transcribed into cDNAs and then applied as templates in PCR analysis. Expression of *Gh_A07G0747* (*GhTOM*) and the NaCl-responsive genes in the transgenic *Arabidopsis* plants was carried out through qRT-PCR analysis with gene-specific primers (Table 1), using the fluorescent intercalating dye FastaStart Universal SYBR-Green Master in a detection system (Roche Diagnostics GmbH, Mannheim, Germany). *Arabidopsis ACTIN2* gene, (*Atactin2*-F (3′-TTGTGCTGGATTCTGGTGATGG-5′) and *Atactin2*-R (3′-CCGCTCTGCTGTTGTGGTG-5′) was used as a standard control in the qRT-PCR reactions. The qRT-PCR mixture preparation was done in accordance with the manufacturer’s instructions. The qRT-PCR reaction mixture was prepared in a total volume of 20 μL, containing 10 μL of SYBR green master mix, 2 μL of cDNA template, 6 μL of deionised H_2_O, and 2 μL of each primer to make a final concentration of 10 μM. The PCR thermal cycling conditions were as follows: 95 °C for 10 min; 40 cycles of 95 °C for 5 s, 60 °C for 30 s, and 72 °C for 30 s. Data were collected during the extension step: 95 °C for 15 s, 60 °C for 1 min, 95 °C for 30 s, and 60 °C for 15 s. Three biological replicates and three technical replicates were performed for each cDNA sample.

### 2.14. RNA Isolation and qRT-PCR Analysis of the Transformed Gene in Cotton Tissues

The EASYspin Plus plant RNA extraction kit obtained from Aid Lab (Beijing, China) was used to extract RNA from two sets of samples under normal conditions and under salt stress treatment. Under normal conditions, RNA was extracted from the roots, stem, sepal, leaf, petal, stamen, pistil, seed, and fibre tissues; this was to determine which tissue exhibited higher upregulation of the transformed gene. In salt treatment, at an NaCl concentration of 300 mM, only leaf tissues were collected for RNA extraction at 0 h, 3 h, 6 h, 12 h, 24 h, 36 h, and finally at 48 h of salt stress exposure. The concentration and quality of each RNA sample was determined through gel electrophoresis and a NanoDrop 2000 spectrophotometer criterion. Only RNAs which met the criterion 260/280 ratio of 1.8–2.1, 260/230 ratio ≥2.0 were used for further analyses and stored at –80 °C. The *Actin7* gene was used as a reference (forward sequence (3′-ATCCTCCGTCTTGACCTTG-5′) and reverse sequence (3′-TGTCCGTCAGGCAACTCAT-5′) and specific *TOM* genes primer (forward sequence (3′-TGCGAAAGCTTTTTCATCATTGG-5′) and reverse sequence (3′-ACTTGTAGACGGGGC TGGTA-5′) were used for qRT-PCR. The first-strand cDNA synthesis was carried out with TranScript-All-in-One First-Strand cDNA Synthesis SuperMix for qRT-PCR, obtained from Transgen Biotech (Beijing, China), used as per the manufacturer’s instructions. Primer Premier 5 [54] was used to design *Gh_A07G0747 (GhTOM)* gene specific primers, with melting temperatures of 55–60 °C, primer lengths of 18–21 bp, and amplicon lengths of 101–221 bp. The Fast Start Universal SYBRgreen Master (Rox) (Roche, Mannheim, Germany), was used to perform qRT-PCR as per the manufacturer’s instructions. Reactions were made in a total volume of 20 μL, containing 10 μL of SYBR green master mix, 2 μL of cDNA template, 6 μL of deionised H_2_O, and 2 μL of each primer to make a final concentration of 10 μM. The qRT-PCR thermal cycling conditions were as follows: 95 °C for 10 min; 40 cycles of 95 °C for 5 s, 60 °C for 30 s, and 72 °C for 30 s. Data were collected during the extension step: 95 °C for 15 s, 60 °C for 1 min, 95 °C for 30 s, and 60 °C for 15 s. Three biological replicates and three technical replicates were performed per cDNA sample.

## 3. Results

### 3.1. Identification, Sequence, and Structure Analysis of TOM Candidate Proteins of G-Protein-Coupled Receptors in Cotton

**Figure 1:**
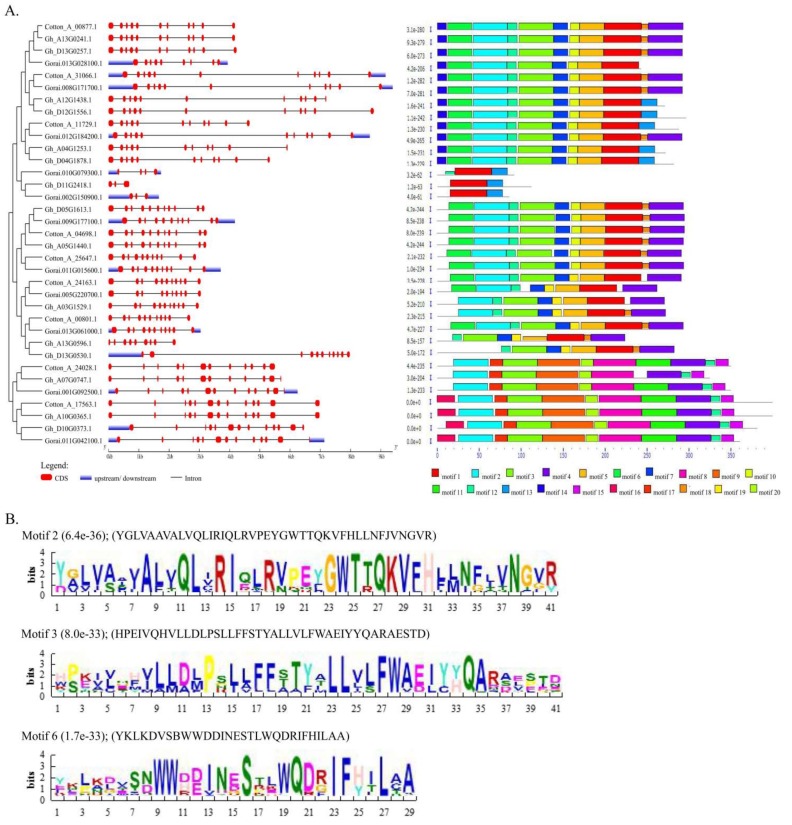
(**A**) Phylogenetic tree, gene structure, and motif of all the cotton *TOM* genes. Red colour indicates the exons, blue indicates the up/downstream, and the grey lines are the introns. The motifs are numbered 1 to 20 and are illustrated by differently-coloured motifs unique to cotton *TOM* genes; (**B**) Common motifs which define the cotton-specific *TOM* gene.

By the use of the functional domain PF06454, we obtained 16 *TOM* gene members in *G. hirsutum* (one of which was scaffold, thus not included in further analysis), 9 in *G. arboreum*, and 11 in *G. raimondii*. The numbers were varied by analysing their respective sequences through Pfam scan and SMART online software tools. The cotton *TOM* genes were basically sub-classified as *TOM1, TOM3* and *THH1*; the highest members were *TOM1* with 20 genes, followed by *TOM3* with 14 genes, while for *THH1* there was only one (Table 2). The physiochemical properties of the TOM proteins were determined. The isoelectric point (PI) values ranged from 6.501 (Gorai.011G042100.1) to 10.33 (Gh_D13G0530.1); protein lengths ranged from 86 amino acids (aa) (Gorai.002G150900.1) to 396 aa (Gh_A10G0365.1 and Cotton_A_17563.1); molecular weights ranged from 10.049 kDa (Gorai.002G150900.1) to 44.279 kDa (Cotton_A_17563.1); grand average of hydropathy (GRAVY) values ranged from 0.337 (Gorai.002G150900.1) to 0.753 (Gorai.010G079300.1) (Supplementary Table S1). The results obtained for these cotton genes are in agreement with previous reports, in which the GPCR isolated from the pea GPCR gene (*PsGPCR*) had a predicted molecular mass of about 35 kDa and PI value of 10.60 [55]. All the genes were interrupted by introns; the highest number on intron interruption was detected in *Gh_D10G0373.1*, while the fewest intron disruptions were detected in *Gh_D11G2418.1* and *Gorai.002G150900.1* (Figure 1 and Supplementary Table S2). The results obtained are in agreement with various studies on the functional analysis of stress genes; for instance, in the functional analysis of *MYB* genes in sesame, a majority were found to be interrupted by introns despite the fact that introns affect the functionality of the genes due to energy demand [56]. Based on motif analysis, the TOM proteins in cotton were functionally identifiable by three distinct motifs, which were found to be common among the three cotton genotypes: motif 2 (YGGRLFFMLRRFPIESKGRRKKLQEVGYVTGICFTCFLVRCIMMCFSAFDKAAD), motif 3 (DHPVLNLIYYMLVEILPSALVLFILRKLPPKRVSNQYHPIR), and motif 6 (WWKYIPVLVIJSKMFIAGVSLFAALGFLL). The cotton TOM proteins contain various proportions of amino acids. Leucine (0.092), serine (0.074), alanine (0.073), glycine (0.069), valine (0.064), and glutamic acid (0.062) were highest in proportion, while histidine (0.022), cysteine (0.018), and tryptophan (0.013) were the least in proportions.

### 3.2. Phylogenetic Analysis and Functional Classification of TOM Candidate Proteins of G-Protein-Coupled Receptors in Cotton 

**Figure 2:**
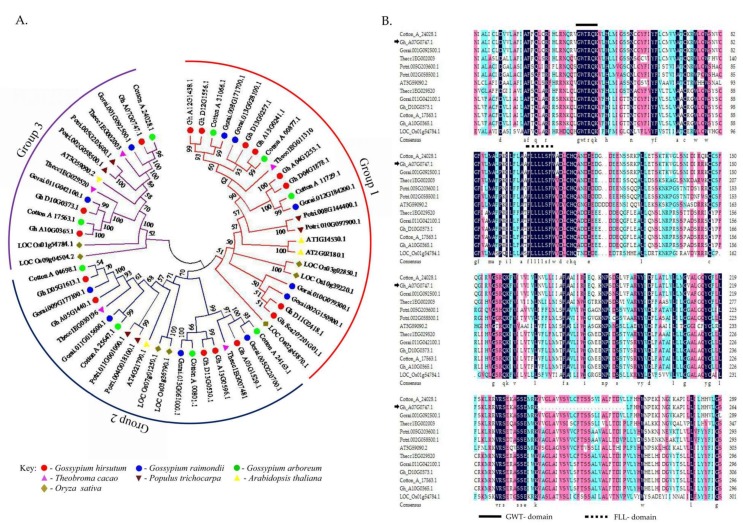
Multiple sequence alignment of Gh_A07G0747 (*GhTOM*) protein and phylogenetic tree analysis. (**A**) Gh_A07G0747 (*GhTOM*) proteins tree analysis built using MEGA 7.0 program together with proteins from other plants as illustrated in the key. (**B**) Amino acids alignment of Gh_A07G0747 (*GhTOM*) with other TOM-GPCRs from other plants: Cotton_A_24028.1; Gorai.001G092500.1; Thecc1EG002003; Potri.005G203600.1; Potri.002G058500.1; AT3G59090.2; Thecc1EG029520; Gorai.011G042100.1; Gh_D10G0373.1; Cotton_A_17563.1; Gh_A10G0365.1 and LOC_Os01g54784.1.

Phylogenetic tree analysis provides a road map of the origin and evolutionary nature of genes [57]. All the determined *TOM* genes from the three cotton genomes (*G. hirsutum*, *G. arboreum*, *G. raimondii*), as well as from *Oryza sativa*, *Populus trichocarpa*, *Theobroma cacao*, and *A. thaliana* were found to be subdivided into three groups based on the phylogenetic tree analysis. No orthologous gene pairs were found between any of the cotton genotypes to their closest relative, *T. cacao*. All the orthologous gene pairs were found between *G. hirsutum* and *G. arboreum*; *G. hirsutum* and *G. raimondii*; and/or *G. arboreum* and *G. raimondii*. A unique observation was made in the orthologous gene pairs between the tetraploid cotton, *G. hirsutum*, to the two diploid parental lineages. In all the orthologous gene pairs, the corresponding pair from diploid cotton was homologous to the parental line of the tetraploid gene; for instance, *Gh_A13G0241.1*-*Cotton_A_00877.1* (99%), *Gh_D13G0257.1*-*Gorai.013G028100.1* (93%), *Gh_A10G0365.1*-*CottonA17563.1* (100%), *Gh_D10G03713.1*-*Gorai.011G042100.1* (99%), and finally *Gh_A07G0747.1*-*Cotton_A_24028.1* (96%). All gene pairs exhibited high percentage similarities; a similar observation was also made among the orthologous gene pairs between the diploid cotton genomes (Figure 2A). In the analysis of the late embryogenesis abundant (*LEA*) genes in cotton in relation to other plants, no orthologous gene pairs were found between the *LEA* genes of cotton to other plants; all orthologous gene pairs were found among the cotton *LEA*s [39].Therefore, the results obtained in this research are in agreement with the previous findings of a number of functional analyses of stress genes in plants.

The alignment results of the Gh_A07G0747 (*GhTOM*) protein with other TOM-GPCR from other plants, Cotton_A_24028.1, Gorai.001G092500.1, Thecc1EG002003, Potri.005G203600.1, Potri.002G058500.1, AT3G59090.2, Thecc1EG029520, Gorai.011G042100.1, Gh_D10G0373.1, Cotton_A_17563.1, Gh_A10G0365.1, and LOC_Os01g54784.1 obtained from *Gossypium raimondii* (Gorai…), *Theobroma cacao* (Thecc…), *Oryza sativa* (LOC…), *Arabidopsis thaliana* (AT…), and *Populus trichocarpa* (Potri…) exhibited high similarities ranging from 52% to 100%. The aligned sequences revealed two types of functional domain which have never been described in any literature (“GWT” and “FLL”), with most of them exhibiting the C-terminal domain (Figure 2B). The detection of the C-terminal domain is in agreement with earlier descriptions of GPCRs by Tuteja, [55], who stated that some GPCRs contain a cysteine residue in the C-terminal domain which serves as the main site for palmitoylation, mainly referring to site for lipid modification.

### 3.3. Chromosome Mapping and Subcellular Localization of the TOM Candidate Proteins of G-Protein-Coupled Receptors Proteins in Cotton

The genes were not mapped in all chromosomes, both in tetraploid and diploid cotton; this could be explained by the number of genes to be mapped in all the cotton chromosomes—15 genes in *G. hirsutum* against the 26 chromosomes (one was scaffold and not used for further analysis), 9 and 11 genes in *G. arboreum* and *G. raimondii*, respectively, against 13 chromosomes of the diploid cottons. The tetraploid *TOM* genes were mapped in 13 chromosomes out of 26 in *G. arboreum*, covering 50% of all the chromosomes (7 out of 13), while in *G. raimondii* it was 9 chromosomes out of 13. This indicates the eminent role played by these genes, as they have a wide coverage of the entire cotton genome despite their low numbers. In relation to the subcellular localization, all the bioinformatics tools used gave similar results, indicating that the *TOM* genes are embedded within the plasma membrane at the cellular level and were evidently involved in secretory pathways (Table 3).

### 3.4. RNA Isolation and qRT-PCR Analysis of the Transformed Gene in Cotton Tissues

**Figure 3:**
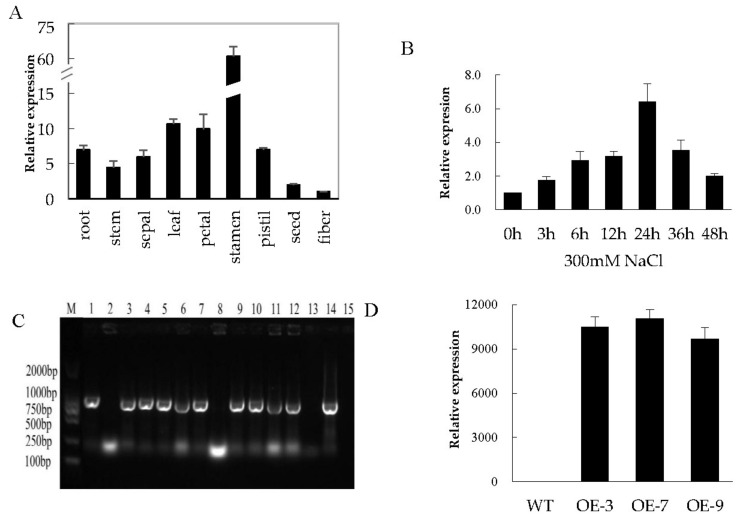
Real-time quantitative reverse transcription polymerase chain reaction (qRT-PCR) analysis of the expression of the transformed gene *Gh_A07G0747* (*GhTOM*)*.* (**A**) Total RNA isolated from two-week-old cotton plant under normal conditions; (**B**) Total RNA extracted from salt-stressed cotton seedlings; (**C**) Polymerase chain reaction (PCR) analysis performed to check 978 bp coding sequence (CDS) integration in transformed T0 generation, number 1–13 transgenic lines, 14 positive control (*pWM101-Gh_A07G0747* (*GhTOM*)), and 15 is the negative control (wild-type, WT). Expression of the transformed genes in transgenic; (**D**) the transcripts levels of the Gh_A07G0747 (GhTOM) of T2 transgenic lines analysed through qRT-PCR.

In order to confirm whether the transformed gene in *Arabidopsis* is present in cotton tissues, we carried out the qRT-PCR analysis of the expression of the gene using RNA samples extracted from different tissues of cotton plant under normal conditions. We found that the gene was more abundantly expressed in the stamen and leaves compared to other tissues analysed (Figure 3). In addition, we carried out treatment on cotton seedlings after the emergence of three true leaves under high salt stress condition (300 mM NaCl); leaf samples were collected for RNA extraction at intervals of 0 h, 3 h, 6 h, 12 h, 24 h, 36 h, and 48 h. A steady increase from 0 h to 24 h followed by a steady decline in gene expression was observed in salt-treated plants.

### 3.5. Analysis of Oxidant and Antioxidant Content in the Transgenic Lines and Wild-Type under Salt Stress Condition

**Figure 4:**
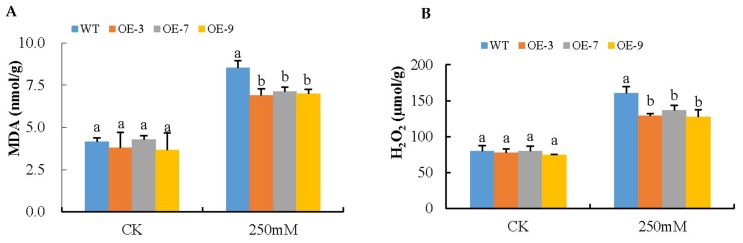
Changes of malondialdehyde (MDA) and H_2_O_2_ in *Gh_A07G0747 (GhTOM)* transgenic lines under salt stress. (**A**) Determination of MDA accumulation in leaves of wild-type (WT) and both overexpressed (OE) lines (OE-3, OE-7, and OE-9) after 8-day salt stress; (**B**) Quantitative determination of H_2_O_2_ accumulation in leaves of WT and both OE lines (OE-3, OE-7, and OE-9) after 8-day salt stress. In (**A**,**B**), each experiment was repeated three times. Bar indicates standard error (SE). Different letters indicate significant differences between wild-type and OE lines (ANOVA; *p* < 0.05). CK: normal conditions.

In the analysis of the oxidants MDA and H_2_O_2_, the *Gh_A07G0747 (GhTOM)* transgenic *Arabidopsis* had significantly lower levels of MDA compared to the wild type under salt stress conditions, implying that the transgenic plants were subjected to significantly reduced oxidative damage compared to the wild type. Because MDA is a measure of chlorophyll damage [58], it was imperative to determine the concentration of reactive oxygen species (ROS) accumulation in both wild-type and the different lines of transgenic model plant under salt stress conditions. The quantities of H_2_O_2_ in all three lines of transgenic plants were significantly lower than the wild-type, as illustrated in (Figure 4). The quantitative determination of H_2_O_2_ was carried out after 8 days of salt stress exposure. The H_2_O_2_ content increased in both WT and transgenic lines after salt stress exposure. However, the transgenic lines OE-3, OE-7, and OE-9 accumulated significantly reduced levels of H_2_O_2_, (only 69.4% increase on average) relative to WT (101.1% increase) after salt stress. Under the controlled experiment, no significant differences were detected in either MDA or H_2_O_2_ between the transgenic lines (OE-3, OE-7, and OE-9) and the wild type (WT). The results obtained clearly show that the transformed gene had a net effect of enhancing salt stress tolerance by significantly reducing the damages caused by ROS.

**Figure 5:**
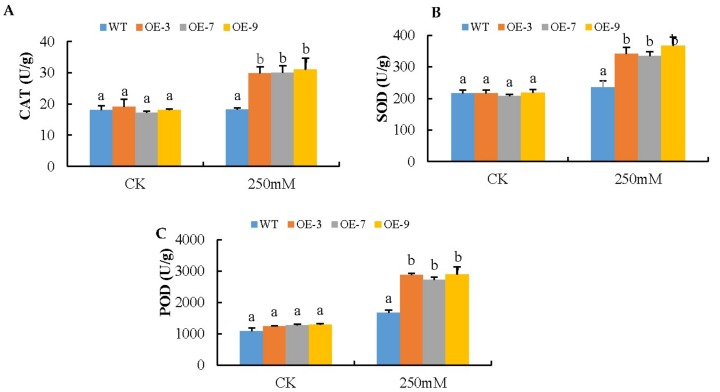
Activities of antioxidant enzymes catalase (CAT), superoxide dismutase (SOD), and peroxidase (POD) in WT and *Gh_A07G0747 (GhTOM)*—transgenic lines. Four weeks old WT and transgenic plants were salt-stressed for 8 days and then the leaf samples were taken and used to detect activities of CAT, SOD and POD. (**A**) CAT activity; (**B**) SOD activity; (**C**) POD activity. Data are means ± SE calculated from three replicates. Different letters indicate a significant difference between the WT and both transgenic lines (ANOVA; *p* < 0.05). Three independent experiments were carried out and the results were similar.

The regulation of reactive oxygen species is carried out by enzymatic oxidants [26]. In lieu of this, we carried out the determination of various antioxidants (POD, CAT, and SOD) in the transgenic lines and the wild type under salt stress conditions. Under control conditions, no significant difference was observed in SOD, CAT, and POD activities between transgenic lines and the wild type (WT). When the plants were exposed to salt stress after 8 days, transgenic lines OE-3, OE-7, and OE-9 exhibited significant differences and high activities of SOD, CAT, and POD compared to the wild type (WT) (Figure 5). The results obtained for the antioxidants and the oxidants enzymes clearly demonstrated that the transformed gene *Gh_A07G0747* (*GhTOM*) had an overall effect on ROS accumulation by inducing more antioxidant activity under salt stress conditions.

### 3.6. Over-Expression of Gh_A07G0747 (GhTOM) in Plants Confers Enhanced Salt Tolerance

**Figure 6:**
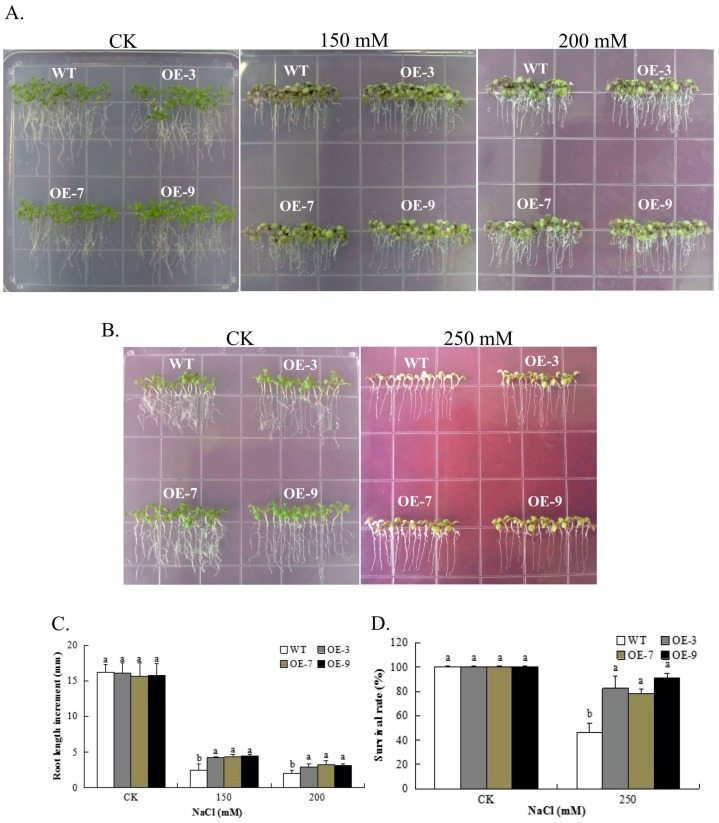
Comparative analysis of *Gh_A07G0747* (*GhTOM*) transgenic and WT plant lines under salt stress conditions. (**A**) *Gh_A07G0747* (*GhTOM*) overexpressing and WT plants were grown vertically in 0.5 Murashige and Skoog (MS) medium supplemented with 0, 150, and 200 mM NaCl and incubated for 6 days. (**B**) Survival rate of transgenic and wild type; (**C**) Comparison of root length between transgenic lines and WT seedlings grown on 0.5 MS medium containing different concentrations of NaCl; (**D**) Comparison of survival rate between transgenic lines and WT seedlings grown on 0.5 MS medium containing different concentrations of NaCl after 4 days of salt stress. Each value is the mean ± SD (*n* = 3), the letters “a” and “b” indicate that there is significant difference between the *Gh_A07G0747* (*GhTOM*) transgenic and the wild-type seeds, which were determined through *t*-tests (* *p* = 0.05).

Plant tolerance to salt stress has been found to be positively correlated with rate of root growth, and therefore primary root growth is an indicator of stress tolerance [59]. We examined the root length and survival rate of the transgenic lines and wild type under salt stress conditions. The *Gh_A07G0747* (*GhTOM*) transgenic lines seedlings showed longer roots and had greener broader rosettes than the wild type under 150 mM NaCl and 200 mM NaCl (Figure 6). The survival rates among the transgenic lines were significantly higher compared to the wild type in 250 mM of NaCl, however under controlled conditions, no significant difference was observed between the transgenic lines and the wild type. The wild type leaves all changed to yellow, with no green pigmentation after 4 days of salt stress exposure (Figure 6). The ability of the transgenic lines to maintain their chlorophyll for a longer period under higher level of salt stress demonstrates that the gene had a significant and positive effect on enhancing salt tolerance in the transgenic lines.

### 3.7. Transcripts Investigation of Salt Stress-Responsive Genes

**Figure 7:**
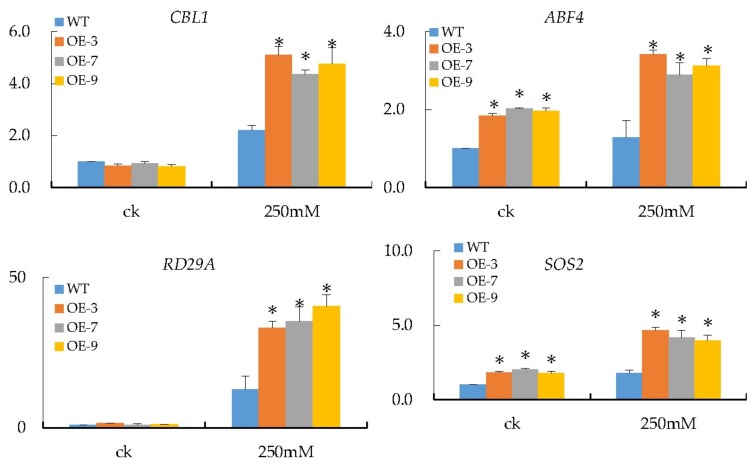
Expression levels of salt stress-responsive genes (*CBL1*, *ABF4*, *RD29A*, and *SOS2*) in wild-type and transgenic lines. *Arabidopsis ACTIN2* was used as the reference gene, mean values with ± SD. * *p* < 0.05 as calculated by Student’s *t*-test.

Several prior studies have recommended numerous good stress-related marker genes. Thus, in this study, we chose *ABF4*, *SOS2*, *CBL1*, and *RD29A* as salt stress-responsive marker genes and checked their transcripts in *Gh_A07G0747 (GhTOM)* and WT transgenic *Arabidopsis* treated with and without 250 mM NaCl for 4 days, in order to dissect the possible regulatory relationships of marker genes with *Gh_A07G0747* (*GhTOM*). The qRT-PCR results showed that the *RD29A* gene exhibited the most significant upregulation compared to the rest, with the highest level of expression at 60 compared to the rest with maximum expression levels ranging from 4 to 6 (Figure 7). 

### 3.8. Analysis of Chlorophyll Content in Gh_A07G0747 (GhTOM) Transgenic Lines

**Figure 8:**
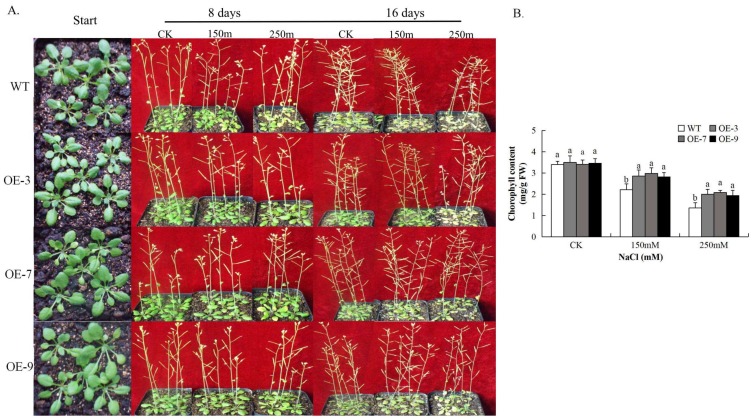
Phenotypes of wild-type (WT) and *Gh_A07G0747* (*GhTOM*)-transgenic Arabidopsis plants in response to salt stress. (**A**) Salt tolerance of potted plants of wild-type and *Gh_A07G0747* (*GhTOM*)—OE *Arabidopsis*. Four-week-old WT and transgenic OE (OE-3, OE-7, and OE-9) plants were grown in soil in pots for 8 and 16 days under salt stress; (**B**) Chlorophyll determination between the wild-type and the transgenic lines after 8 days of salt stress. Values are mean ± SE, *n* = 12, and significant differences between wild-type and OE lines are indicated by different letters.

The ability of the plant to carry out photosynthesis under salt stress conditions depends on its ability to regulate ROS production being released from respiring cells [60]. We determined the level of chlorophyll content on transgenic and wild type plants. Under normal conditions, *Gh_A07G0747* (*GhTOM*) lines and WT showed no clear significant differences in chlorophyll content, but after 16 days, salt-stressed samples OE-3, OE-7, and OE-9 had significantly higher contents of chlorophyll as shown in (Figure 8). So, these significant levels in overexpressed transgenic plants strengthen the speculation that because of less damage to the photosynthetic apparatus, transgenic plants maintained a stay-green property, thus distinguishing its role in salt stress tolerance.

### 3.9. Subcellular Localization of Gh_A07G0747 (GhTOM) Protein

**Figure 9:**
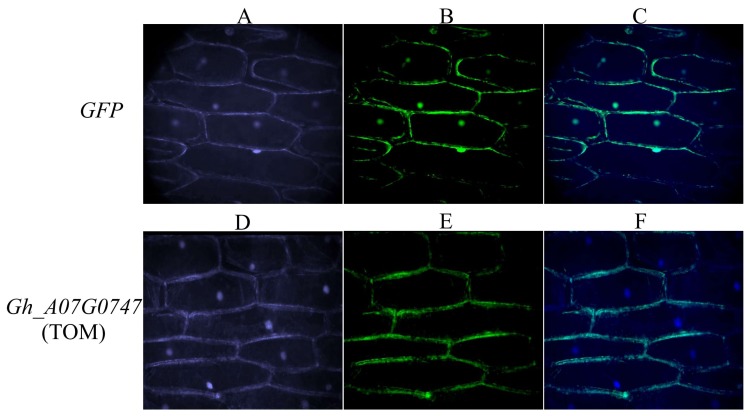
Localization of Gh_A07G0747 (*GhTOM*) in onion epidermal cells. (**A**–**C**) Onion epidermal cells transformed with 35S::GFP; (**D**–**F**) Onion epidermal cells transformed with 35S::*GFP–Gh_A07G0747* (*GhTOM*). (**A**,**D**) Light field with magnification of X400 to display morphology. (**B**,**E**) Dark field images for the detection of green fluorescent protein (GFP) fluorescence. (**C**,**F**) Superimposed light and dark field images.

To investigate the subcellular localization of Gh_A07G0747 (GhTOM), we fused the *Gh_A07G0747* (*GhTOM*) gene in frame with GFP and transiently expressed this gene in onion epidermal cells. Previous work reported that the *TOM* genes are membranous and the same was predicted before using various bioinformatics tools. Thus, in order to determine the cellular location of Gh_A07G0747 (GhTOM), a *GFP–Gh_A07G0747* (*GhTOM*) fusion vector (pBI121-*Gh_A07G0747* (*GhTOM*)) driven by the 35S promoter was constructed and transformed into LBA4404 cells. As a negative control, transgenic LBA4404 cells expressing only GFP were used. As a positive control, transgenic LBA4404 cells expressing GFP*–Gh_A07G0747* (*GhTOM*) was confirmed to be localized at the plasma membrane (Figure 9). The detection of the gene at the plasma membrane validated the previous findings and the results obtained from bioinformatics analysis. The plasma membrane is greatly affected by salt toxicity, affecting the osmotic balance and membrane stability [61].

## 4. Discussion

Among the most significant abiotic stresses with huge economic losses in plant productivity is salt stress [62]. The problem of salt stress is currently estimated to affect over 6% of the entire arable land globally [63]. Therefore, the only probable solution to drastically reduce the effect of salt stress in plants and improve productivity is by developing plant genotypes with enhanced tolerance to salinity. A number of studies have pointed out various stress tolerance genes with profound effects in enhancing abiotic tolerance in plants. Among them are members of G-protein-coupled receptor (GPCR) genes, in which *TOM* is a sub-family member. The relationship of *TOM* genes to GPCR is debatable, though various studies have shown that *TOM1* and the distant homologs *CAND3*, *CAND4*, and *CAND5* are candidate plant GPCRs [64]. TOM is known to belong to the domain of unknown function (DUF1084) with PF06454. A number of members of proteins which fall within the domain of unknown functions such as DUF143 have been found to be critical in ribosomal silencing mechanism, which prevents the synthesis of ribosomes, which in turns enhances the cells’ adaptability to low nutrient levels by seizing protein biosynthesis [65]. The proteins found to be members of the domain of unknown function have been found to be critical in both plants and animals when the cells are under starvation. The main cause of starvation in cells is mainly triggered by abiotic and biotic stresses. 

When plants are exposed to salt stress, the photosynthetic apparatus shuts down or is greatly reduced, affecting the normal function of the plant [66]. In this research work, we explored the possible roles of *TOM* genes in enhancing salt stress in plants by carrying out critical analysis of the abundance of these gene sub-families in the three cotton genomes. It was interesting to note that despite the fact that the main gene family, GPCR, has not been widely studied in plants as compared to animals, we were able to detect various members of *TOM* genes in varying proportions (16 genes in *G. hirsutum*, 9 in *G. arboreum*, and 11 in *G. raimondii*)*,* the quantities of these genes in tetraploid cotton (AD) clearly indicated that these genes had undergone a period of gene loss or the genes had possibly acquired new functions even though they evolved through whole genome duplication (WGD) in the tetraploid cotton. Based on the various physiological characteristics of these members, the *TOM* genes were found to have low molecular weights ranging from 10.049 kDa to 44.279 kDa, with all their GRAVY values of less than one an indication that these proteins were hydrophobic in nature [67]. The identity of *TOM* genes and the hydrophobic nature is coherent with a number of salt stress tolerance genes such as *CsRCI2A* and *CsRCI2E*, which were isolated from *Camelina sativa* (L.) (a biodiesel crop with high tolerance to cold stress); the two genes were found to be highly induced due to salinity stress [68].

The tobamovirus_1 (*TOM1*) gene is a critical gene which is required for the efficient multiplication of tobamoviruses, positive-strand RNA viruses infecting a wide variety of plants [69]. Although the gene is associated with a wide array of viruses, a study conducted on the variation levels of oxidant and antioxidant enzymes in compatible and incompatible hosts showed increased levels of peroxidase, salicylic acid, lipid peroxidation, protein oxidation, H_2_O_2_, and decreased CAT in incompatible host–tobamovirus interaction compared to compatible hosts [70]. The ability of the *TOM1* gene to have a regulatory role under salt stress indicates that it is a functional member of the GPCR genes. In this study, the transformed plants exhibited significantly reduced levels of oxidant enzymes under salt stress and increased levels of antioxidants such as CAT, POD, and SOD. Salt stress causes injury to the plasma membrane as a result of an increased production of reactive oxygen species [71]. The ability of the transgenic plant to induce more of the antioxidant enzymes demonstrates the signalling cascades played by the *Gh_A07G0747* (*GhTOM*) gene in plants under salt stress conditions. In addition, increased production of ROS leads to increased oxidative stress, causing peroxidation of membrane lipids, which leads to increased levels of MDA within the plant cell [72]. In the analysis of MDA concentration between the transgenic and wild-type groups, there was a significant reduction in the levels of MDA in transgenic lines compared to the wild type, which demonstrated that the transgenic lines suffered less damage under salt stress conditions. Similar results have also been obtained in transformed *Arabidopsis* plants with cotton gene such as *GHSOS1*; the level of oxidant enzymes were found to be significantly reduced in the transgenic lines as compared to the wild-type [73].

We carried out an expression analysis of the transgenic lines together with wild types. Gene expression profiles provide valuable information for determining the possible functions or action of the genes under stress conditions [74]. The transformed cotton gene was detected in all tissues in varying proportions, but was significantly abundant in stamen, leaf, and petal, in which we encourage further research in order to elucidate the exact role played the transformed gene in these organs. Reproduction is necessary for the continuity of the plant; thus, the ability to produce viable seeds is a survival strategy of many plants under extreme conditions. The stamen and petals are components of the reproductive organs, and could possibly aid in the fertilization and development of viable seeds. The expression levels of the various candidate genes of GPCRs are known to be highly correlated with abiotic stress tolerance [22,23,75,76,77,78]. In the present study, there was a significant difference in the expression levels of *Gh_A07G0747* (*GhTOM*) in the leaves under salt treatment, but levels were extremely low in wild-type under the same condition. The limitation of plant growth by abiotic stress cannot be directed to a single physiological process. The dominant physiological process is photosynthesis. Plant growth as biomass production is a measure of the overall net photosynthesis, and therefore, abiotic stresses affecting growth also affect photosynthesis. Salt stress causes either short or long-term effects on photosynthesis. The leaf is the primary organ for photosynthesis; the photosynthetic pathways are enzymatically controlled and pH sensitive. Salt stress inhibits photosystem 2 (PS2) activity—PS2 primarily mediates non-cyclic electron transport from water to plastoquinone (PQ) [79]. The higher expression of the transformed gene could possibly aid in the maintenance of the photosystem 2 mechanism under salt stress conditions. In addition, salt-sensitive plants have been found to have low chlorophyll concentration. For instance, chlorophyll contents have been found to be significantly reduced in in salt-susceptible plants such as tomato [80] and potato [81], among other crops. The opposite has been observed among salt tolerant plants, which have been found to exhibit higher chlorophyll concentration (e.g., in pearl millet) [82]. To investigate if *Gh_A07G0747* (*GhTOM*) affects the expression of salt stress genes in plants, we used four salt genes which have been extensively studied in modal plants, *ABF4*, *SOS2*, *CBL1*, and *RD29A*, and analysed their expression levels in the *Gh_A07G0747* (*GhTOM*) transgenic *Arabidopsis* lines OE-3, OE-7, and OE-9, as well as the wild type. The results indicated that the expression levels of *ABF4*, *SOS2*, *CBL1*, and *RD29A* genes were significantly higher in the transgenic plant lines, as compared to the wild type under salt stress conditions. The calcineurin B-like protein1 (CBL1) has been shown to be a member of the calcineurin B-like calcium sensor proteins family, and has been found to interact with CBL-interacting protein kinase 23 (CIPK23). The *CBL1* genes could possibly be acting as a regulatory subunit of a plant calcineurin-like activity controlling the calcium signalling pathways under certain salt stress conditions. The *CBL1* expression level has been investigated and was found to be highly increased under various abiotic stress factors, such as high salinity, cold, and water deficit [83]. Calcineurin B-like proteins (CBLs) are mainly members of Ca^2+^ ion sensor proteins that mainly interact with a group of serine/threonine kinases denoted as CBL-interacting protein kinases (CIPKs) to form complexes known as calcineurin B-like proteins_CBL-interacting protein kinases (CBL-CIPK) [83,84]. CBL–CIPK complexes are mainly involved in relaying plant responses to many environmental signals (e.g., salt, drought), and are also involved in the regulation of ion fluxes [85,86,87]. The salt gene, *ABF4* (ABRE-binding factor 4) is a transcription factor which mainly interacts with ABA (abscisic acid) responsive elements (ABREs) and primarily functions as transcriptional activator in ABRE-dependent transcription in *Arabidopsis* [88]. The upregulation of *ABF4* in *Arabidopsis* has been found to respond to ABA treatment, drought, and salinity stress [89]. A number of studies have shown that *RD29A* (responsive to desiccation 29A) and *RD29B* (responsive to desiccation 29B) are induced by conditions such as dehydration, low temperature, high salinity, or by treatment with exogenous ABA. The induction of the two genes occurs because the promoter regions of *RD29A* contains the cis-acting dehydration-responsive element (DRE) while *RD29B* possesses two ABA-responsive elements (ABREs) in its promoter regions, which are essential for their expression under stress condition [90,91]. 

In this investigation, we found that overexpression of *Gh_A07G0747* (*GhTOM*) enhanced the expression levels of all four salt genes *(ABF4*, *SOS2*, *CBL1*, and *RD29A*) in the transgenic plants under normal growth conditions and under salt stress conditions, which gave an indication that *Gh_A07G0747* (*GhTOM*) had a regulatory role in the transgenic plant and could possibly be functioning as regulatory transcriptome in enhancing salt stress tolerance in transgenic *Arabidopsis*. 

Primary plant root growth under salt condition is known to be one of the phenotypic traits of salt tolerance genotypes [92]. The transgenic plants exhibited longer roots compared to the wild type under salt stress conditions; the enhanced root lengths improved their capacity for increased water intake and conduction of water up the plants [93]. We were able to locate G*h_A07G0747* (*GhTOM*) in the plasma membrane. The plasma membrane is an important organelle responsible for maintaining osmotic balance within the plant [94]. The gene could possibly be responsible for maintaining the cell membrane integrity under salt stress conditions. Salt stress has been found to cause the main primary photosynthetic organelle to be highly disorganized; the number and size of plastoglobuli increase, and their starch content decreases [95]. In the mesophyll of sweet potato leaves, chloroplast thylakoid membranes have been found to be swollen, and most are lost under severe salt stress [96]. Therefore, the localization of the transformed gene within the plasma membrane could be of great significance in regulating the injuries caused as result of salt toxicity, by either increasing the level of antioxidant enzymes or through Na^+^ ion extrusion and transportation from roots to the shoots, a similar role to one which has been found to be played by the salt stress tolerance enhancing cotton gene known as *GhSOS1*, a plasma membrane Na^+^/H^+^ antiporter gene [73].

## 5. Conclusions

We identified a total of 36 genes in all three cotton genomes, 16 of *G. hirsutum*, 9 *G. arboreum*, and 11 genes of *G. raimondii.* All genes were found to be disrupted by introns. However, the mean intron lengths of the genes ranged from 172.5 to 788.8, while the mean exon lengths ranged from 70.8 to 200.2. It was found that the introns affected the functionality of the genes due the high energy demand during the splicing [97]. However, most of the stress-related genes have intron interruptions. Therefore, the expression of the genes cannot be affected by the intron disruption alone—the intron–exon ratio could possibly be playing a major role in regulating the process of gene expression. TOM1 is a unique protein, and is a member of proteins of unknown function (domain of unknown function). Its ability to confer salt stress tolerance in transgenic *Arabidopsis* could be a breakthrough, even though it has been reported that proteins of unknown functions are mainly mobilized when cells are faced with starvation. The results obtained in this research provide a solid foundation for the exploration of the various members of the GPCRs in plants, for their possible role in enhancing abiotic and biotic stress tolerance. The GPCRs have been intensively studied in animals, but limited studies have been done in plants. To date, no consensus has been reached on the various members of the plant GPCRs. Several investigations have provided conflicting information on whether TOM1 and their distant homologous similar proteins such as CAND3/4/5 are truly members of the plant GPCRs [98]. Their key position in cellular signal transduction means that GPCRs act as critical regulators. Moreover, the fact that their dysfunction can result in several diseases has made GPCRs the targets of the majority of all currently prescribed drugs [8]. It seems that the signals are mostly perceived at the level of membrane, and therefore transmembrane events are the likely routes for signal generation and transduction. The low level of oxidants such as MDA and H_2_O_2_ and increased levels of antioxidants such as CAT, SOD, and POD in the overexpressed lines OE-3, OE-7, and OE-9 compared to the wild type clearly indicate the significant role played by the novel gene *Gh_A07G0747* (*GhTOM*) in enhancing salt tolerance in *Arabidopsis*. In addition, the chlorophyll content was significantly higher in the transgenic lines, which indicated that the genes had a greater contributory role in maintaining low-level oxidations and maintenance of the photosystem 2 process of the photosynthetic pathways within the plant. Based on the results obtained in this research work, we infer that the transformed genes, which are a member of GPCRs, can be exploited further in order to understand the exact roles in various plant biochemical and physiological pathways in enhancing tolerance to salinity.

## Figures and Tables

**Table 1:** Gene specific primers used in real-time quantitative reverse transcription polymerase chain reaction (qRT-PCR) analysis for the expression of the salt genes in the transgenic *Arabidopsis* plant.

Genes	Forward Sequence	Reverse Sequence
*AtABF4*	3′-AACAACTTAGGAGGTGGTGGTCAT-5′	3′-TGTAGCAGCTGGCGCAGAAGTCAT-5′
*AtSOS2*	3′-GGCTTGAAGAAAGTGAGTCTCG-5′	3′-GCTACATAGTTCGGAGTTCCACA-5′
*AtCBL1*	3′-GAAATGAAACTGGCTGATGAAACCATAGAG-5′	3′-CTCGTGGCAATCTACTCGGTCTTAAACC-5′
*AtRD29A*	3′-TGAAAGGAGGAGGAGGAATGGTTGG-5′	3′-ACAAAACACACATAAACATCCAAAGT-5′

**Table 2:** The candidate G-protein-coupled receptors (GPCRs) in *Gossypium hirsutum*, *Gossypium arboreum*, and *Gossypium raimondii*. The identification of the GPCR candidate genes and annotation done as described by Gookin [18].

Gene Type	Prediction	Family	Sub Family	Sub Family Type	*Gossypium hirsutum*	*Gossypium arboreum*	*Gossypium raimondii*
*TOM1*	GPCR	class A	Olfactory	Olfactory II family 10	8	6	6
*TOM3*	GPCR	class A	Olfactory	Olfactory II family 5	7	3	4
*THH1*	GPCR	class A	Peptide	-	0	0	1
Total					15	9	11

**Table 3:** Subcellular localization of the cotton *TOM* candidate genes of G-protein-coupled receptors in cotton.

Gene ID	Gene Name	Chromosome Locations			TargetP						Pprowler	Wolfpsort
		Chr.	Start	End	Len	cTP	mTP	SP	other	Loc		
*Gh_A03G1529*	*TOM1*	A03	95,951,060	95,954,035	271	0.022	0.339	0.129	0.743	_	SP	plas
*Gh_A04G1253*	*TOM3*	A04	62,591,785	62,597,706	272	0.025	0.028	0.107	0.986	_	OTHER	plas
*Gh_A05G1440*	*TOM1*	A05	14,950,490	14,953,714	291	0.016	0.051	0.433	0.952	_	SP	plas
*Gh_A07G0747*	*TOM1*	A07	11,687,587	11,693,293	325	0.003	0.052	0.924	0.044	S	SP	plas
*Gh_A10G0365*	*TOM1*	A10	3,340,797	3,347,772	396	0.003	0.056	0.751	0.59	S	SP	plas
*Gh_A12G1438*	*TOM3*	A12	73,175,985	73,183,181	268	0.019	0.035	0.176	0.986	_	OTHER	plas
*Gh_A13G0241*	*TOM3*	A13	2,823,366	2,827,544	289	0.006	0.075	0.218	0.981	_	OTHER	plas
*Gh_A13G0596*	*TOM1*	A13	14,111,783	14,113,994	224	0.013	0.153	0.191	0.98	_	SP	plas
*Gh_D04G1878*	*TOM3*	D04	51,121,020	51,126,349	282	0.021	0.031	0.114	0.987	_	OTHER	plas
*Gh_D05G1613*	*TOM1*	D05	14,554,705	14,557,876	291	0.014	0.059	0.387	0.956	_	SP	plas
*Gh_D10G0373*	*TOM1*	D10	3,299,454	3,305,915	377	0.007	0.228	0.116	0.967	_	SP	plas
*Gh_D11G2418*	*TOM3*	D11	48,310,923	48,311,606	112	0.017	0.406	0.232	0.41	_	SP	plas
*Gh_D12G1556*	*TOM3*	D12	46,513,482	46,522,251	293	0.018	0.033	0.177	0.987	_	OTHER	plas
*Gh_D13G0257*	*TOM3*	D13	2,477,467	2,481,698	289	0.006	0.081	0.181	0.983	_	OTHER	plas
*Gh_D13G0530*	*TOM1*	D13	6,994,712	7,002,690	282	0.142	0.054	0.219	0.771	_	SP	plas
*Cotton_A_00801*	*TOM1*	Chr13	71,968,383	71,971,076	272	0.011	0.263	0.368	0.621	_	SP	plas
*Cotton_A_00877*	*TOM3*	Chr13	73,444,587	73,448,764	290	0.005	0.078	0.229	0.978	_	OTHER	plas
*Cotton_A_04698*	*TOM1*	Chr10	20,294,407	20,297,646	292	0.014	0.059	0.387	0.956	_	SP	plas
*Cotton_A_11729*	*TOM3*	Chr07	106,242,296	106,246,957	288	0.025	0.028	0.117	0.985	_	OTHER	plas
*Cotton_A_17563*	*TOM1*	Chr09	89,683,765	89,690,744	396	0.003	0.056	0.751	0.59	S	SP	plas
*Cotton_A_24028*	*TOM1*	Chr01	107,338,814	107,344,313	346	0.003	0.052	0.924	0.044	S	SP	plas
*Cotton_A_24163*	*TOM1*	Chr05	48,844,703	48,847,741	291	0.01	0.07	0.264	0.931	_	SP	plas
*Cotton_A_25647*	*TOM1*	Chr09	74,805,749	74,808,633	291	0.016	0.096	0.34	0.953	_	SP	plas
*Cotton_A_31066*	*TOM3*	Chr06	58,856,809	58,865,968	289	0.017	0.037	0.169	0.987	_	OTHER	plas
*Gorai.001G092500*	*TOM1*	Chr01	10,292,446	10,298,692	350	0.003	0.052	0.924	0.044	S	SP	plas
*Gorai.002G150900*	*TOM3*	Chr02	29,604,352	29,606,009	86	0.012	0.477	0.068	0.448	M	mTP	cyto
*Gorai.005G220700*	*TOM1*	Chr05	60,350,693	60,353,743	262	0.014	0.091	0.299	0.92	_	SP	plas
*Gorai.008G171700*	*TOM3*	Chr08	44,664,816	44,674,208	289	0.019	0.034	0.156	0.988	_	OTHER	plas
*Gorai.009G177100*	*TOM1*	Chr09	13,701,332	13,705,507	292	0.014	0.059	0.387	0.956	_	SP	plas
*Gorai.010G079300*	*TOM3*	Chr10	11,503,072	11,504,804	92	0.009	0.4	0.436	0.05	S	SP	extr
*Gorai.011G015600*	*TOM1*	Chr11	1,095,851	1,099,556	291	0.018	0.061	0.407	0.907	_	SP	plas
*Gorai.011G042100*	*TOM1*	Chr11	3,118,826	3,125,957	357	0.003	0.056	0.751	0.59	S	SP	plas
*Gorai.012G184200*	*TOM3*	Chr12	35,143,300	35,151,941	289	0.021	0.033	0.086	0.989	_	OTHER	plas
*Gorai.013G028100*	*THH1*	Chr13	2,095,047	2,098,979	243	0.007	0.079	0.173	0.984	_	OTHER	plas
*Gorai.013G061000*	*TOM1*	Chr13	6,622,078	6,625,118	293	0.013	0.16	0.274	0.833	_	SP	plas

Abbreviations: Len, sequence length; cTP, a chloroplast transit peptide; mTP, a mitochondrial targeting peptide; SP, a signal peptide; S, secretory pathway; M, mitochondrion; plas, plasma membrane; cyto, cytoplasm; extr, extracellular organelles; chr, chromosome; Loc, Location.

## Supplementary Materials

The following are available online at http://www.mdpi.com/2073-4425/9/4/209/s1. Table S1: Physiochemical properties of the TOM proteins of the GPCR family. Table S2: Intron-exon and their average values for the *TOM*s for the three cotton genomes. Figure S1: RNA expression profile of the cotton *TOM* candidate genes of GPCR functional domain.
