# Supplementary material for: A Novel G-Protein-Coupled Receptors Gene from Upland Cotton Enhances Salt Stress Tolerance in Transgenic Arabidopsis
Source: Genes (Basel). 2018 Apr 12;9(4):209. doi: 10.3390/genes9040209 (PMC5924551; doi:10.3390/genes9040209)
Supplement: Supplementary file 1 [file genes-09-00209-s001.zip › Supplementary materials/Supplementary Table 1.docx]

Supplementary Table 1: Physiochemical properties of the TOM proteins of the GPCR family

| Gene ID | Protein Length (aa) | Molecular Weight (kDa) | Charge | Isoelectric Point | Grand Average of Hydropathy |
| --- | --- | --- | --- | --- | --- |
| Gh_A03G1529 | 271 | 31.478 | 12.5 | 9.352 | 0.579 |
| Gh_A04G1253 | 272 | 31.521 | 3.5 | 7.574 | 0.599 |
| Gh_A05G1440 | 291 | 33.687 | 9 | 9.041 | 0.661 |
| Gh_A07G0747 | 325 | 37.084 | 23 | 10.006 | 0.35 |
| Gh_A10G0365 | 396 | 44.238 | 3 | 7.122 | 0.464 |
| Gh_A12G1438 | 268 | 30.921 | 5.5 | 8.207 | 0.609 |
| Gh_A13G0241 | 289 | 33.603 | 18 | 10.185 | 0.58 |
| Gh_A13G0596 | 224 | 25.927 | 12.5 | 9.84 | 0.627 |
| Gh_D04G1878 | 282 | 32.851 | 5 | 7.992 | 0.576 |
| Gh_D05G1613 | 291 | 33.705 | 9 | 9.041 | 0.652 |
| Gh_D10G0373 | 377 | 42.767 | 2 | 7.04 | 0.351 |
| Gh_D11G2418 | 112 | 12.826 | 6 | 8.494 | 0.565 |
| Gh_D12G1556 | 293 | 34.201 | 8 | 8.858 | 0.422 |
| Gh_D13G0257 | 289 | 33.635 | 17 | 10.105 | 0.552 |
| Gh_D13G0530 | 282 | 32.837 | 23.5 | 10.33 | 0.37 |
| Cotton_A_00801 | 272 | 31.901 | 13.5 | 9.436 | 0.636 |
| Cotton_A_00877 | 290 | 33.688 | 17 | 10.119 | 0.593 |
| Cotton_A_04698 | 292 | 33.776 | 9 | 9.041 | 0.656 |
| Cotton_A_11729 | 288 | 33.516 | 7.5 | 8.815 | 0.617 |
| Cotton_A_17563 | 396 | 44.279 | 4 | 7.385 | 0.433 |
| Cotton_A_24028 | 346 | 39.151 | 21 | 9.645 | 0.504 |
| Cotton_A_24163 | 291 | 33.785 | 14.5 | 9.719 | 0.576 |
| Cotton_A_25647 | 291 | 33.248 | 5.5 | 8.198 | 0.662 |
| Cotton_A_31066 | 289 | 33.456 | 11.5 | 9.546 | 0.6 |
| Gorai.001G092500 | 350 | 39.59 | 23 | 9.858 | 0.479 |
| Gorai.002G150900 | 86 | 10.049 | 6 | 8.838 | 0.337 |
| Gorai.005G220700 | 262 | 30.485 | 17 | 10.016 | 0.499 |
| Gorai.008G171700 | 289 | 33.462 | 11.5 | 9.546 | 0.588 |
| Gorai.009G177100 | 292 | 33.833 | 9 | 9.041 | 0.638 |
| Gorai.010G079300 | 92 | 10.504 | 4.5 | 8.753 | 0.753 |
| Gorai.011G015600 | 291 | 33.382 | 8.5 | 8.872 | 0.698 |
| Gorai.011G042100 | 357 | 40.321 | 0 | 6.501 | 0.432 |
| Gorai.012G184200 | 289 | 33.67 | 9 | 9.044 | 0.574 |
| Gorai.013G028100 | 243 | 28.437 | 12.5 | 9.965 | 0.595 |
| Gorai.013G061000 | 293 | 34.287 | 15 | 9.636 | 0.548 |

aa: amino acid; kDa:kilo Dalton
