# Supplementary material for: A Novel G-Protein-Coupled Receptors Gene from Upland Cotton Enhances Salt Stress Tolerance in Transgenic Arabidopsis
Source: Genes (Basel). 2018 Apr 12;9(4):209. doi: 10.3390/genes9040209 (PMC5924551; doi:10.3390/genes9040209)
Supplement: Supplementary file 1 [file genes-09-00209-s001.zip › Supplementary materials/Supplementary Table 2.docx]

Supplementary Table 2: Intron-exon and their average values for the TOMs for the three cotton genomes

| Gene ID | Transcript Length (bp) | CDS Length (bp) | CDS GC Content (%) | Exon Number | Mean Exon Length (bp) | Mean Intron Length (bp) |
| --- | --- | --- | --- | --- | --- | --- |
| Gh_A03G1529 | 816 | 816 | 41.3 | 11 | 74.2 | 216 |
| Gh_A04G1253 | 819 | 819 | 44.9 | 11 | 74.5 | 510.3 |
| Gh_A05G1440 | 876 | 876 | 42.4 | 11 | 79.6 | 234.9 |
| Gh_A07G0747 | 978 | 978 | 40.6 | 12 | 81.5 | 429.9 |
| Gh_A10G0365 | 1,191 | 1,191 | 40.6 | 13 | 91.6 | 482.1 |
| Gh_A12G1438 | 807 | 807 | 44 | 11 | 73.4 | 639 |
| Gh_A13G0241 | 870 | 870 | 42.1 | 11 | 79.1 | 330.9 |
| Gh_A13G0596 | 675 | 675 | 43 | 9 | 75 | 192.1 |
| Gh_D04G1878 | 849 | 849 | 43.6 | 11 | 77.2 | 448.1 |
| Gh_D05G1613 | 876 | 876 | 42.1 | 11 | 79.6 | 229.6 |
| Gh_D10G0373 | 1,134 | 1,134 | 39.8 | 14 | 81 | 409.8 |
| Gh_D11G2418 | 339 | 339 | 35.4 | 3 | 113 | 172.5 |
| Gh_D12G1556 | 882 | 882 | 43.4 | 11 | 80.2 | 788.8 |
| Gh_D13G0257 | 870 | 870 | 42.3 | 11 | 79.1 | 336.2 |
| Gh_D13G0530 | 849 | 849 | 42 | 12 | 70.8 | 648.2 |
| Cotton_A_00801 | 819 | 819 | 42.2 | 11 | 74.5 | 187.5 |
| Cotton_A_00877 | 870 | 870 | 42.4 | 11 | 79.1 | 330.8 |
| Cotton_A_04698 | 879 | 879 | 42.3 | 11 | 79.9 | 236.1 |
| Cotton_A_11729 | 864 | 864 | 43.6 | 11 | 78.5 | 379.8 |
| Cotton_A_17563 | 1,191 | 1,191 | 40.9 | 13 | 91.6 | 482.4 |
| Cotton_A_24028 | 1,038 | 1,038 | 40.7 | 12 | 86.5 | 405.6 |
| Cotton_A_24163 | 876 | 876 | 40.4 | 11 | 79.6 | 216.3 |
| Cotton_A_25647 | 873 | 873 | 41.8 | 11 | 79.4 | 201.2 |
| Cotton_A_31066 | 1,818 | 870 | 44 | 11 | 165.3 | 734.2 |
| Gorai.001G092500 | 1,701 | 1,053 | 41 | 13 | 130.8 | 378.8 |
| Gorai.002G150900 | 353 | 261 | 36 | 3 | 117.7 | 173.5 |
| Gorai.005G220700 | 789 | 789 | 40.1 | 10 | 78.9 | 251.3 |
| Gorai.008G171700 | 2,030 | 870 | 44 | 11 | 184.5 | 736.3 |
| Gorai.009G177100 | 1,522 | 879 | 42 | 11 | 138.4 | 229.8 |
| Gorai.010G079300 | 518 | 279 | 39.4 | 5 | 103.6 | 303.8 |
| Gorai.011G015600 | 1,695 | 876 | 41.3 | 11 | 154.1 | 201.1 |
| Gorai.011G042100 | 1,827 | 1,074 | 39.9 | 13 | 140.5 | 442.1 |
| Gorai.012G184200 | 1,557 | 870 | 44 | 11 | 141.5 | 708.5 |
| Gorai.013G028100 | 1,802 | 732 | 43.6 | 9 | 200.2 | 266.4 |
| Gorai.013G061000 | 1,191 | 882 | 41.7 | 11 | 108.3 | 185 |
